# Reviewers and awards

**DOI:** 10.1093/ehjdh/ztaf098

**Published:** 2025-08-19

**Authors:** Nico Bruining, Robert van der Boon, Isabella Kardys, Paul Cummins, Joost Lumens

**Affiliations:** Department of Cardiology, Erasmus MC, Dr Molewaterplein 40 Rotterdam 3015 GD, The Netherlands; Department of Cardiology, Erasmus MC, Dr Molewaterplein 40 Rotterdam 3015 GD, The Netherlands; Department of Cardiology, Erasmus MC, Dr Molewaterplein 40 Rotterdam 3015 GD, The Netherlands; Department of Cardiology, Erasmus MC, Dr Molewaterplein 40 Rotterdam 3015 GD, The Netherlands; CARIM School for Cardiovascular Diseases, Maastricht University Medical Center, Maastricht, The Netherlands

Peer reviewing is one of the cornerstones of scientific publishing, and we could not be more grateful to each and every one of you who so generously dedicated your time and expertise to support us at the *European Heart Journal—Digital Health (EHJ-DH)*. Your thoughtful feedback helped the authors to improve their work and ensured that our journal maintained the highest standards of quality and impact.

We know that finding time for peer review is not easy. It is often a real challenge amidst busy schedules or even in your spare time. That is why your commitment means so much to us. Your reviews have been instrumental not only in guiding our editorial decisions but also in helping authors improving their manuscripts. Thanks to your efforts, the scientific quality of our publications has grown tremendously.

And we are celebrating: EHJ-DH has achieved again a fantastic milestone with our new ‘impact factor of 4.4’, keeping us in the ‘top quartile’ of cardiovascular journals! This achievement is the also the result of your dedication, strengthening our community. It brought us to our shared ambition, to be, and to stay, the leading journal in cardiovascular digital health.

On behalf of the entire editorial team: ‘thank you’ for your invaluable contributions. We truly couldn’t do it without you!

**Figure 1:**
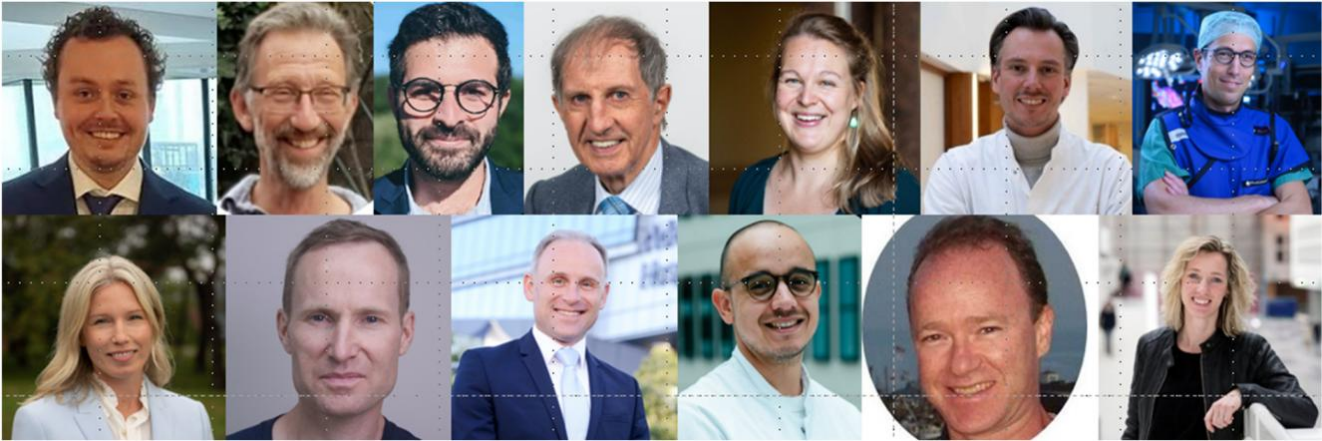
The top reviewers 2024–25: starting at the top left with Niels Scholte, followed by Johan de Bie at the right, Aaram Omar Khader, Peter MacFarlane, Machtheld Boonstra, Mark Schuuring and David Duncker. Bottom row left to right, Emma Svennberg, Erwan Donal, Jeroen Hendriks, Robert van der Boon, Richard Gregg and Sanne Hoeks.

Some numbers: in the past year, almost 600 reviewers helped us in the evaluation of the manuscripts we received. A special thanks go to our Top Reviewers, 13 this year, with the top ranking led by Dr. Niels Scholte, at the top left (*Figure 1*).

Below in alphabetical order the names of all our reviewers for the *European Heart Journal—Digital Health* over the past year. In case we would have forgotten someone by an unfortunate mistake, please accept our apology for this unintentional omission!

**Thank you all so much!**

*The Editorial team.*

Jaime Aboal

Ana Abreu

Piergiuseppe Agostoni

J Airaksinen

Souhila Aittigrine

Oguz Akbilgic

Murat Akcakaya

Ibrahim Akin

Manuel Almeida

Gonzalo Luis Alonso Salinas

Martin Altreuther

Giuseppe Andò

Mohammed Yusuf Ansari

Charalambos Antoniades

Kazutaka Aonuma

Taku Asano

Solmaz Assa

Riccardo Asteggiano

Patricio Astudillo

Shaul Atar

Steven Atlas

Douwe Atsma

Zachi I Attia

Alberto Avolio

Robert Avram

Sara Baart

Erdi Babayiğit

Yong-Soo Baek

Esmée A. Bakker

Andrea Baragetti

Adrian Baranchuk

Arantxa Barandiaran Aizpurua

Rogier Barendse

Joseph Barker

Stanisław Bartuś

Ladislav Batalik

Axel Bauer

Marta Belmonte

Theresa Bender

Niv Ben-Shabat

Peter J. Bentley

Paola Berchialla

Alexander E. Berezin

Philippe Bertrand

Konstanze Betz

Balu Bhasuran

Yochai Birnbaum

Richard Body

Jasper Boeddinghaus

Eric Boersma

Niccolò Bonini

Stephanie Erika Bonn

Machteld Boonstra

Julian Borges

Giuseppe Boriani

Christos V. Bourantas

Zeineb Bouzid

Vladimír Boža

Peter Bramlage

Axel Brandes

Emmanuel Bresso

Salvatore Brugaletta

Nico Bruining

Leszek Bryniarski

Ricardo Budde

Garrett S. Bullock

Thilo Burkard

Aurélien Bustin

Enrico Caiani

Andrea Calandrino

Peter Calvert

Claudio Capelli

Romain Capoulade

Blase A. Carabello

Stéphane G. Carlier

Oren Caspi

Alvin Chandra

Neal A. Chatterjee

Debatri Chatterjee

Hao-Min Cheng

D Chew

Mattia Chiesa

Chi Keong Ching

Youngjin Cho

Eue-Keun Choi

Dong-Ju Choi

J Choi

Celine Chui

James J. Cimino

Tufan Çınar

Matthijs Cluitmans

Gary Collins

Karin Coninx

J Coughian

Dragos Cozma

Philip Croon

Paul Cummins

Pedro Silva Cunha

Jonathan Cunningham

Anne Curtis

Antoine Da Costa

Luis Augusto Palma Dallan

Giuseppe D'Ancona

Fabrizio D'Ascenzo

Shreyasi Datta

Luciano De Biase

Johan de Bie

Jorg de Bruin

John de Heide

PPT de Jaegere

José M. De la torre Hernández

Andrea De Lorenzo

Karina De Santis

Guus A. de Waard

Parastoo Dehkordi

Lukas R.C. Dekker

Michiel Delesie

Wijnand K. den Dekker

Corstiaan den Uil

Rajat Deo

Milind Y. Desai

Jouke Dijkstra

Polychronis Dilaveris

Birthe Dinesen

Daixin Ding

Torsten Doenst

Florian Doldi

Helena Dominguez

Erwan Donal

Jianzeng Dong

Kefei Dou

Philipp Douschan

Ines Drenjancevic

Chong-Yang Duan

Dariusz Dudek

David Duncker

Son Duong

Daniel Dürschmied

Johan Engdahl

Sandy Engelhardt

Cetin Erol

Ian Everitt

Lizhou Fan

Xiaohan Fan

Peter Farjo

Charles Fauvel

Sebastian Feickert

QiPing Feng

Caleb Ferguson

Francisco Fernández-Avilés

Daniel Ferreira

Simone Fezzi

Nenad Filipovic

Scott D. Flamm

Samah Fodeh

Alan Yean Yip Fong

Paul Forsyth

Alan Fraser

Victor F. Froelicher

Yutaka Furukawa

Lebo Gafane-Matemane

Konstantinos A. Gatzoulis

Monika Gawałko

Anil K. Gehi

Arnar Geirsson

Simonetta Genovesi

Robert-Jan van Geuns

Olivier Gevaert

Samad Ghaffari

Yazan Gharaibeh

Noemi Giordano

Nicolas Girerd

Renata Główczyńska

Su-Gang Gong

Nieves Gonzalo Lopez

Wilson W. Good

Shinichi Goto

j goudzwaard

Felice Gragnano

Austin Graybeal

Richard E. Gregg

Jean-Marie Grégoire

Sophie Gu

Marco Guazzi

Michael Guckert

Yutao Guo

Yingkun Guo

Anubha Gupta

Mohit D. Gupta

Michelle Gurvitz

Przemysław Guzik

Saskia Haitjema

Bernhard Hametner

Eileen M. Handberg

Tine Willum Hansen

Alexander Harrison

Juha Hartikainen

Christian Hassager

Ward Heggermont

Martin Hemels

Jeroen Hendriks

Yasutomi Higashikuni

Shungo Hikoso

Sanne Hoeks

Shenda Hong

Amin Hossein

James Howard

Chieh-Chun Huang

John Hughes

Heikki V. Huikuri

Rebecka Hultgren

Chung-Lieh Hung

Moritake Iguchi

Nobuhiro Ikemura

Jacopo Francesco Imberti

Omer Inan

Sheikh Mohammed Shariful Islam

Toshiaki Isogai

Edward Itelman

Kazuhiro P. Izawa

Rod Jackson

Vincent Jacquemet

Amir Jadidi

Sun-Joo Jang

Yuheng Jia

Haibo Jia

Haibo Jia

Yanrui Jin

Jens Brock Johansen

Ethan Johnson

Andrew D. Johnson

Louisa Jorm

Christian Jung

Tsunekazu Kakuta

Hidehiro Kaneko

Si-Hyuck Kang

Soo-Jin Kang

Jørgen K. Kanters

Suraj Kapa

Ibrahim Karabayir

Lina Karout

Sazzli Kasim

Yoshinori Katsumata

Clifford J. Kavinsky

Yoshiaki Kawase

Yuhe Ke

Daniel Keene

H Kelbaek

Thomas W. Kelsey

Hareld Kemps

Hareld M. C. Kemps

Nurgel Keser

Alexander Kharlamov

Shaan Khurshid

Daehoon Kim

Justin Namuk Kim

Jin Kirigaya

Takeshi Kitai

Sverre E. Kjeldsen

Neal S. Kleiman

Leonie Klompstra

Sébastien Knecht

Ryoung-Eun Ko

Satoshi Kodera

Friedrich Koehler

Chaitanya Kolluru

Łukasz Kołtowski

Dimitrios Konstantinidis

Attila Kónyi

Ran Kornowski

Grigorios Korosoglou

Attila Kovács

Peter R. Kowey

Rob Krams

Vessela Krasteva

Karl-Patrik Kresoja

Jose E Krieger

Philipp Krisai

Sridhar Krishnan

David E. Krummen

Bartosz Krzowski

Stefan Kulnik

Akira Kurata

Sudhir Kurl

Kenya Kusunose

Jennifer Kwan

Soonil Kwon

Bhone Myint Kyaw

Konstatinos Kyriakoulis

Gokce Banu Laleci Erturkmen

Pablo Lamata

Ekaterini Lambrinou

Sandra Lauck

Michael Leasure

Hyeonhoon Lee

Ki Hong Lee

Hak-Seung Lee

Chan Joo Lee

Seunghwa Lee

Paul Leeson

Mattie Lenzen

Matthew A. Levin

Susanne Lezius

Lingyao Li

Gen-Min Lin

Daniel Lindholm

Gregory Lip

Stephen Little

Tianyi Liu

Guangzhi Liu

Axel Loewe

Juan Miguel Lopez Alcaraz

Francisco Lopez-Jimenez

Nabaouia Louridi

Pernille Lunde

Alexander Maass

Peter Macfarlane

Guy A. MacGowan

Andrew Mackie

Akiko Maehara

Julien Magne

Edris Mahtab

Lars S. Maier

Hisaki Makimoto

Sorayya Malek

Abdulaziz Malik

Marek Malik

Alessandro Maloberti

m mamprin

Martin Manninger

Konstantinos Margetis

Eloi Marijon

Francisco Marín

Maria Marketou

Nassir Marrouche

Mauro Massussi

Ioannis Mastoris

Hitoshi Matsuo

Francesco Mattace Raso

Adam M. May

Christopher C. Mayer

Pamela J. McCabe

Robert L. McNamara

Manuel Meier

Wouter Meijers

Felipe Meneguitti Dias

Munib Mesinovic

David Messika zeittoun

Olivier Meste

Anastasia Mihailidou

Bogdan Milicevic

Robert Miller

Rafael N Miranda

Sayan Mitra

Suneet Mittal

Takuya Mizukami

Tomohiro Mizuno

Philip Moons

Cristian Mornos

Bobak J. Mortazavi

Stephan Mueller

Tobias Mühling

Dirk Müller-Wieland

Patricia Munroe

Tadashi Murai

Evan Muse

Marius Myrstad

Hafiz Naderi

Mayooran Namasivayam

Abdulqadir J. Nashwan

Uyên Châu Nguyên

Stephen J. Nicholls

Josef Niebauer

Vlasis Ninios

Nobuhiro Nishii

Stefano Nistri

Migue Nobre Menezes

Naoki Nonaka

Gabrielle Norrish

Evangelos Ntalianis

Rutger-Jan Nuis

Wesley O’Neal

Takeyuki Oba

Kevin O'Gallagher

Evangelos Oikonomou

Takahiro Okumura

Hiroyuki Okura

Laura Olivieri

Aaram Omar Khader

Stefano Omboni

Minoru Ono

Alexander R. Opotowsky

Leopoldo Ordine

Declan Oregan

Michele Orini

Nuria Ortigosa

Hui-Nam Pak

Valeria Pannunzio

Michail Papafaklis

Andreas S. Papazoglou

Kosmas I. Paraskevas

Gianfranco Parati

Duk-Woo Park

Seung-Jung Park

Niels Peek

Pierpaolo Pellicori

Carlos Peña-Gil

Marco V. Perez

Nicholas Peters

Steffen Petersen

Ricardo Petraco

Jan Piek

Eva Piotrowicz

Laurent Pison

Nikki A H A Pluymaekers

Katrina Poppe

Evgenij Potapov

Sandra Prescher

Gregg S. Pressman

Jiří Přibil

Nicola Pugliese

Kuberan Pushparajah

Frank Rademakers

Sholeh Rahman

Zahra Raisi-Estabragh

Sanjay Rajagopalan

Julia Ramirez

Tomasz Rechciński

James A. Reiffel

Claire Ren

Joanna Ribeiro

Antonio Luiz Ribeiro

Janik Riese

Hyungtaek RIM

Albert Rogers

Ivan Rokos

Giulio Francesco Romiti

Eelko Ronner

Thomas Rostock

Wolfgang Rottbauer

Cynthia L. Russell

Elisabetta Salvioni

Annett Salzwedel

José Alberto San Román

Anja Sandek

Veer Sangha

Motohiro Sano

Vincenzo Ezio Santobuono

Tabinda Sarwar

Arunashis Sau

Roderick W.C. Scherptong

Martijn Scherrenberg

Todd T. Schlegel

Douglas D. Schocken

Paul Schoenhagen

U. Joseph Schoepf

Niklas Schofer

Niels Scholte

Richard Schuessler

Tobias Schupp

Mark Schuuring

Ojasav Sehrawat

Raja Selvaraj

Partho Sengupta

Arnold H. Seto

Ian Shadforth

Isaac Shiri

Smit Shrivastava

Georgios Sianos

Rosa Sicari

BS. Sidhu

Jennifer Silva

Joao Silva Marques

John Simpson

Arvind Singhal

Konstantinos Siontis

Helen Sjöland

Ioannis Skalidis

Piotr Slomka

Stephen Smith

Marjolein Snaterse-Zuidam

Anna C. Snavely

Tsunenari Soeda

Jiangping Song

Nils Arne Sörensen

Erin M. Spaulding

Sebastian Spethmann

Parvathaneni Naga Srinivasu

Stavros Stavrakis

Benjamin Steinberg

Ralph Stewart

Michael J. Stirratt

Kiril M. Stoyanov

Marc Strik

Jordan B. Strom

Collin M. Stultz

Md. Abu Sufian

Zhonghua Sun

Emma Svennberg

Cees Swenne

Mariusz Szymanski

Silke Szymczak

Stefano Taddei

Masaaki Takeuchi

Isabella Tan

İbrahim Halil Tanboğa

Kouhyar Tavakolian

Yannick Taverne

Gabriel Tensol Rodrigues Pereira

James T. Teo

Nienke Ter Hoeve

Dominic Theuns

Shahane Tigranyan

Jani Tikkanen

Roland R. Tilz

Jitto Titus

Fleur Tjong

Márton Tokodi

R Tolsma

Naoko Tomitani

Aaron Trask

Matthias Trenner

Roderick Treskes

John Triedman

Konstantinos Tsioufis

Shengxian Tu

Karam Turk-Adawi

Raphael Twerenbold

Daiju Ueda

Saraschandra Vallabhajosyula

Rutger van de Leur

Rutger van de Leur

Gert van den Berg

Robert van der Boon

Niels van der Sangen

Willeke van der Stuijt

Enno van der Velde

Linda van Laake

Mathijs van Schie

Bert Vandenberk

Panos Vardas

Niraj Varma

Vassilios Vassiliou

Antti Vehkaoja

Ashwin Venkateshvaran

Johan W. Verjans

Jeroen Vervalcke

Rafael Vidal-Perez

Francesco Vitali

Konstantinos Vlachos

Amit N. Vora

Thien Vu

Reza Wakili

Jonathan Waks

Yinglong Wang

Ji-Guang Wang

Tzung-Dau Wang

Jerremy Weerts

Ramsey Wehbe

Florian Wenzl

Jelmer Westra

Sip Wijchers

Brandon M. Wiley

Michiel M. Winter

Jianhua Wu

Ying Wu

Grégoire Wuerzner

Joanna J. Wykrzykowska

Tianyu Xiang

Meng Xu

Jeong Hoon Yang

Gang Yang

Zonghai Yao

Yan Yao

John Yearwood

Taishi Yonetsu

Yeonyee Yoon

Hee Tae Yu

Kun Yu

Doron Zahger

Junaid A. B. Zaman

Nasibeh Zanjirani Farahani

Katarina Zeder

Hajo Zeeb

Tanja Zeller

Michael J. Zellweger

Jiayin Zhang

Jichao Zhao

Hanjun Zhao

Zhe Zheng

Jiandong Zhou

Alexander Zimmermann

Alex Zwanenburg

David Zweiker

Peter-Paul Zwetsloot

